# Differential Motor Neuron Impairment and Axonal Regeneration in Sporadic and Familiar Amyotrophic Lateral Sclerosis with SOD-1 Mutations: Lessons from Neurophysiology

**DOI:** 10.3390/ijms12129203

**Published:** 2011-12-09

**Authors:** Tommaso Bocci, Chiara Pecori, Elisa Giorli, Lucia Briscese, Silvia Tognazzi, Matteo Caleo, Ferdinando Sartucci

**Affiliations:** 1 Unit of Neurology, Department of Neuroscience, Pisa University Medical School, Pisa 56126, Italy; E-Mails: tommaso.bocci@live.it (T.B.); chiara.pecori@hotmail.com (C.P.); elisa.giorli@alice.it (E.G.); lu.bri@hotmail.it (L.B.); tognozzi86@interfree.it (S.T.); 2 Department of Neuroscience, Neurology and Clinical Neurophysiology Section, University of Siena, Siena 53100, Italy; 3 CNR Neuroscience Institute, Pisa 56124, Italy; E-Mail: caleo@in.cnr.it; 4 Department of Neuroscience, SD of Neurology, Cisanello Hospital, Pisa University Medical School, Pisa 56124, Italy

**Keywords:** Amyotrophic Lateral Sclerosis, SOD-1 carriers, macro-EMG, MUNE

## Abstract

Amyotrophic Lateral Sclerosis (ALS) is a degenerative disorder of the motor system. About 10% of cases are familial and 20% of these families have point mutations in the Cu/Zn superoxide dismutase 1 (SOD-1) gene. SOD-1 catalyses the superoxide radical (O^−2^) into hydrogen peroxide and molecular oxygen. The clinical neurophysiology in ALS plays a fundamental role in differential diagnosis between the familial and sporadic forms and in the assessment of its severity and progression. Sixty ALS patients (34 males; 26 females) were enrolled in the study and examined basally (T0) and every 4 months (T1, T2, and T3). Fifteen of these patients are SOD-1 symptomatic mutation carriers (nine males, six females). We used Macro-EMG and Motor Unit Number Estimation (MUNE) in order to evaluate the neuronal loss and the re-innervation process at the onset of disease and during follow-up period. Results and Discussion: SOD-1 mutation carriers have a higher number of motor units at the moment of diagnosis when compared with the sporadic form, despite a more dramatic drop in later stages. Moreover, in familiar SOD-1 ALS there is not a specific time interval in which the axonal regeneration can balance the neuronal damage. Taken together, these results strengthen the idea of a different pathogenetic mechanism at the base of sALS and fALS.

## 1. Introduction

Amyotrophic Lateral Sclerosis (ALS) is a clinically and genetically heterogeneous, late-onset, neurodegenerative disorder of the motor system [1]. Five to ten percent of cases are familial and about 20% of these families have point mutations in the Cu/Zn superoxide dismutase 1 (SOD-1) gene [2]. The familial form is indistinguishable from the sporadic one with a mean age at clinical onset of 50 years. Mutations in Cu/Zn superoxide dismutase (SOD-1) have stimulated a huge amount of interest. More than 100 missense mutations in SOD-1 have been identified to date [3]. The age of onset of ALS in SOD-1 mutation carriers is variable, beginning from the second decade [4,5]. Superoxide dismutase (SOD-1) is a well characterized enzyme, which exists as a homodimer whose sequence of 153 amino acids is remarkably well conserved across species. However, the pathogenic mechanisms underlying disease’s induction in familiar cases are still largely controversial. The major hypothesis is that familiar ALS, SOD-1 positive, could be caused by a neuronal damage, due to a gradual accumulation of a toxic product SOD-1 derived; this cumulative damage may be due to oxidative stress, leading to a disruption of the cytoskeleton and organelle trafficking within motor neuron dendrites. As the amount increases, a critical threshold may be reached, which overwhelms cellular homeostasis, resulting in fast cell death [6,7]. Aggregates do not exclusively occur in neurons, but also in glial cells, raising the question of whether mutant SOD-1 expression in neurons is sufficient *per se* to induce pyramidal degeneration and sustain disease evolution over time [8–10]. Another intriguing explanation is that disulfide cross-linking and the ensuing formation of SOD-1 aggregates represent a secondary event in the pathogenetic cascade, following either a delayed maturation of intramolecular disulfide bonds or the induction of conformational abnormalities [11]. For instance, in G93A mice, about 40% of the motor endplates denervate at 50 days approximately, three months before the appearance of significant SOD-1 aggregation [12], suggesting that neuron-specific expression of mutant superoxide dismutase is sufficient to induce cortico-spinal degeneration [13,14]. The SOD-1 mutation was initially thought to reduce the neurons protection from oxidation, but a weight of evidence now suggests that the mutated enzyme has a toxic gain of function [15]. Although different mutations in SOD-1 have different effects on the progression of the disease once symptoms occur, the different mutations do not influence the age of clinical onset [16]. It has therefore been suggested that there may be a long preclinical period of motor neuron loss before the onset of symptoms [10,17].

In the absence of a biological marker to establish diagnosis, electrodiagnostic tool (EDX) in ALS plays a critical role in the diagnosis as well as in the assessment of its severity and progression [18–20]. EMG investigation, usually performed with concentric needle electrodes, represents the gold standard [21–23]. Amplitude, duration, area, shape, stability on repeated discharges of Motor Unit Potentials (MUPs), and activity at full effort are parameters conventionally used to evaluate the disease’s stage [19,24]. A particular method to record the full MU potentials is the so-called macro-EMG [25,26]. This technique provides information from a larger area of the muscle than the needle EMG methods. That represents a quantitative neurophysiology technique and can be applied to follow progress and effects of putative therapies [22], or to assess the size of individual motor unit (MU) [27,28]. Among EDX techniques, the methodology of Motor Unit Number Estimation (MUNE) has often been previously employed in assessing loss of surviving MU in ALS [29–33]. Compared with previous studies [21,22], our innovatory idea was to take into consideration simultaneously macro-EMG and MUNE changes in both proximal and distal muscles in the same sample of patients with a 1-year follow-up, to assess whether fALS and sALS differ from each other in terms of motor unit loss and changes.

## 2. Results and Discussion

### 2.1. Motor Unit Number Estimation (MUNE)

Values of MUNE and Macro-EMG parameters are provided in Table 1.

**Figure 1:**
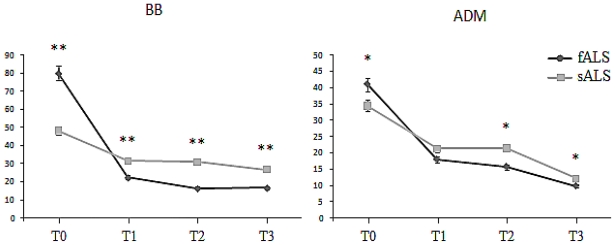
MUNE values for biceps brachialis (BB, on the left) and abductor digiti minimi muscles (ADM, on the right). Note that, at the moment of diagnosis, motor units number is higher for familiar cases (black lines, fALS) compared with sporadic ones (gray lines, sALS); as the disease progresses, motor unit loss becomes more pronounced in the first group. Moreover, compared with sporadic form, patients carrying SOD-1 mutations did not show evidence of partial recovery within eight months from clinical onset (* *p* < 0.05; ** *p* < 0.01).

MUNE values (Figure 1) in ALS patients were below normal limits in 55 (91.7%) and within normal limits in 5 (8.3%) in biceps brachialis (BB) muscle; in 58 (96.7%) and in 2 (3.3%) in abductor digiti minimi (ADM) muscle, respectively [32]. Functioning MUs number progressively decreased in both muscles throughout the entire follow-up period. The Pearson’s correlation coefficient was 0.80, suggesting the rate and amount of MU decrease was approximately similar in both muscles [34]. In ALS, MUNE exhibited a parallel trends in proximal and distal muscles (BB and ADM), independently of disease duration; mean step area, instead, increased more in BB, especially in patients with longer disease duration. The MUNE’s results as concerns patients with fALS, SOD-1 positive, were 80.2 ± 7.8 (T0), 21.8 ± 2.2 (T1), 16.8 ± 1.0 (T2) and 16.5 ± 2.2 (T3) for BB and 42.8 ± 6.6 (T0), 18.4 ± 3.1 (T1), 15.3 ± 2.1 (T2) and 9.0 ± 2.1 (T3) for ADM. Curiously, SOD-1 fALS patients showed a higher number of functioning motor units in the early stage of disease (*p* < 0.001) and a more dramatic drop in later phases (Figure 1). These results suggest a normal pool of motor units in asymptomatic familiar ALS carriers [35]. No electrodiagnostic difference was found between patients with different SOD-1 point mutations.

**Figure 2:**
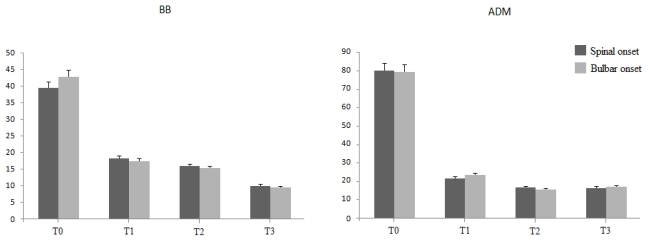
Histogram showing MUNE values in both BB (left) and ADM (right) muscles at every time of follow-up, in spinal (dark gray) and bulbar-onset (gray) cases of fALS. Note the lack of any significant difference between spinal ad bulbar forms for both proximal and distal muscles, throughout the entire follow-up period.

**Figure 3:**
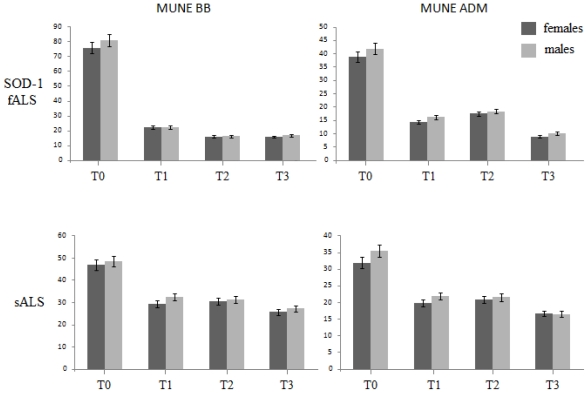
Histogram highlighting MUNE values in both BB (left) and ADM (right) muscles at every time of follow-up, in females (black columns) and males (gray columns); the top row shows the evolution of motor unit loss in the familiar form, whereas the bottom one the trend in sporadic cases. The lack of significant differences between males and females, in sporadic as well as in familiar forms, is consistent with results recently reported by Hegedus and colleagues (see the text for a more exhaustive discussion).

Moreover, we did not found any significant difference between spinal and bulbar-onset fALS in terms of surviving MU, for both BB and ADM muscles (*p* > 0.05, Figure 2), as well as between males and females (*p* > 0.05, Figure 3).

### 2.2. Macro-EMG

**Figure 4:**
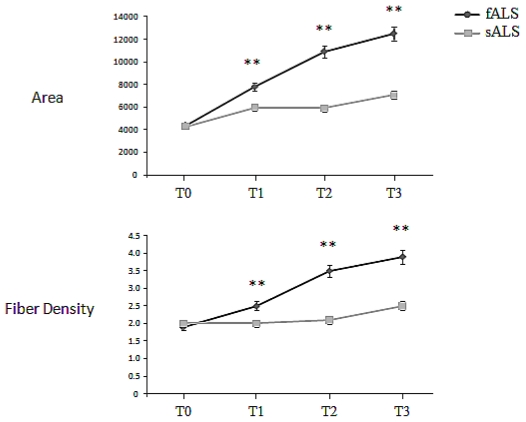
Time trend of Macro-EMG parameters (area, fiber density) with time. All the values increase more steeply in familiar than in sporadic forms (black and gray lines, respectively), strengthening the idea that in the first group there is a paradoxical more effective axonal sprouting (* *p* < 0.05; ** *p* < 0.01).

In sALS patients at T0, both Macro-motor Unit Potentials (Macro-MUPs) area and fiber density (FD) were above upper normal limits (Figure 4; for a global overview of Macro-EMG in healthy subjects, see Sartucci *et al*. [32,33]): macro-MUP area was 4397.6 ± 255.9 μVms, mean FD 2.0 ± 0.2 (a summary of results is given in Figure 2). The macro EMG MUP area was abnormal in 57 (95.0%) and normal in 3 (5.0%) patients; in SOD-1 carriers baseline values of MUP area and FD matched with those of sALS patients (4378.9 ± 319.6 μVms and 1.9 ± 0.3, for Macro-MUPs area and FD respectively; *p* = 0.815 and *p* = 0.147). In sALS, Macro-MUPs area resulted progressively increased at every time, especially at T3, compared with T0 (Figure 4): Area: +45.3% (T1); +49.0% (T2); +83.6% (T3); FD showed a trend to increase up to T3: +3.5% (T1); +15.4% (T2); +22.4% (T3). Interestingly, in SOD-1 carriers there was a much steeper increase at T1, T2 and T3 in respect to sporadic forms, as concerns both Macro-MUPs area and FD values. Macro-MUPs area was 7791.0 ± 953.4, 10922.8 ± 1123.7 and 12499.3 ± 1874.4 (*p* < 0.01) μVms and mean FD 2.5 ± 0.3, 3.5 ± 0.6 and 3.9 ± 0.5 (*p* > 0.01). Taken together, these results account both for a more severe involvement of alfa-motorneurons pool and a paradoxical more effective axonal sprouting in fALS compared with sALS.

## 3. Experimental Section

### 3.1. Patients and Methods

Family members of known SOD-1 positive families were contacted and informed of this study. In the group of 15 symptomatic SOD-1 mutation carriers, two were found to have a point mutation in exon 4, codon 100, GAA to GGA—Glu100Gly; two were found to have a point mutation in exon 4, codon 113, ATT to ACT—Ile113Thr; five were found to have a point mutation in exon 5, codon 148, GTA to GGA—Val148Gly.; and six with homozygous for aspartate-to alanine mutations in codon 90 (homD90A), representing the most common SOD-1 mutation with a typical recessive fashion inheritance. Sixty ALS patients (34 males: mean age ± SD 60.0 ± 15.5 years, range 20–82; 26 females: mean age ± SD 62.0 ± 9.2 years, range 30–82) were enrolled in the study and examined basally (T0) and every 4 months (T1, T2, and T3). Fifteen of these patients are familial (SOD-1 mutation carriers, 9 males: mean age ± 1 SD 46.3 ± 14.8 years, range 20–68; 6 females: mean age ± 1 SD 49.0 ± 8.5 years; range 30–65). Macro Motor Unit Potentials (macro-MUPs) were derived from Biceps Brachialis (BB) muscle; MUNE was performed both in BB and Abductor Digiti Minimi (ADM) muscles of the same side. Thirty-three healthy volunteers (13 females and 20 males, mean age: 57.7 ± 13.8 years, range 28–77 years) served as controls. All patients had probable or definite ALS, according to the criteria of the World Federation of Neurology [19]. The sample group of patients included cases with a disease duration from clinical onset of symptoms to the time of the first examination less than 48 months (mean ± SD: 12.2 ± 11.0 months); only few cases had disease duration less than this limit (11 patients; about 14.3%). Twenty-two patients presented a bulbar onset and the remaining a spinal one. Regarding symptoms and signs, in carriers SOD-1 mutation, 10 patients have the spinal type, while only 5 patients have the bulbar type. Although different mutations in SOD-1 have different effects on the progression of the disease once symptoms occur, the different mutations do not influence the age of onset of symptoms. Forty patients were treated with riluzole (Rilutek^®^, 50 mg) at a mean daily dosage of 100 mg (50 mg BID) throughout the entire period of EDX follow-up. Both patients and controls gave their written informed consent prior to participation in the study that had been approved by the local Ethic Committee and followed the tenets of Helsinki.

### 3.2. Macro-EMG

Standard macro-EMG method was applied [28]. We employed a recording electrode, consisting of a modified single fibre EMG (SFEMG) electrode with the cannula Teflon insulated except for the distal 15 mm. The SFEMG recording surface was exposed 7.5 mm from the tip and the recording was made using two channels: the first one in whom the SFEMG activity was displayed (using the cannula as reference) and used to identify the MU and trigger the averaging procedure (band-pass filter for this channel: 500–10 KHz); fiber density (FD) of the triggering single fibre electrode was recorded. The second channel averaged the activity from the cannula until a smooth baseline and a constant macro MUP was obtained (Filter pass-band: 5–10 KHz).

We measured from the averaged signal the total area between the curve and the baseline, the maximal peak-to-peak amplitude (macro-MUP) during the total sweep time of 70 ms [36]. Results were expressed as individual area values from at least 20 recordings. The relative macro amplitude was expressed as the obtained mean value [28]. Fibre density was expressed as number of time locked spikes obtained on the SFEMG channel [37].

In 29 patients (subgroup 1, SG1: 19 males and 10 females; mean age ± 1 SD: 60.0 ± 11.8 years; range 30–78 years; spinal/bulbar onset: 22/7; mean disease duration 29.7 months) macro EMG was repeated after 4 months (T1). Among the second subgroup, 11 patients (subgroup 2, SG2: 8 males and 3 females; mean age ± SD: 57.0 ± 12.8 years; range 30–72 years; spinal/bulbar onset: 10/1; mean disease duration 31 months) were re-tested after 8 months (T2) and in 8 (Subgroup 3, SGP3; 7 males and 1 female; mean age ± SD: 58.0 ± 13.6 years; range 31–82 years; spinal/bulbar onset: 7/1; mean disease duration 37 months) after 12 months from the first examination.

### 3.3. Motor Unit Number Estimation (MUNE)

MUNE technique was performed on the same Keypoint^®^ EMG equipment (Medtronic Dantec, Copenhagen, Denmark) provided with specific software (version 3009) for data acquisition and processing at same time and immediately after macro EMG on the same test session [38]. The used technique relayed on manual incremental stimulation of the motor nerve, known as the McComas technique [37], modified by Ballantyne and later implemented by Stålberg. The following test settings were used: sweep duration 50 ms, gain 2 mV/Div for M wave, 0.5 mV/Div for each step; filters 20–10 KHz [38]. The use of specific software for MUNE detects “alternation”, eliminates subjectivity and the sampling of artifactually small motor units in ALS patients [39,40]; ten incremental steps were recorded [32].

Percutaneous stimuli were delivered over musculocutaneous nerve immediately below axilla, recording from BB muscles, and ulnar nerve at the wrist by recording from the ADM muscle of the same upper limb [32]. Signals were detected with common surface electrodes, Ag/AgCl type, tapered on the cutis over target muscles with a common muscle-belly tendon montage. In those patients who underwent follow-up after 4, 8 and 12 months, each test was performed exactly on the same side with the same electrode position (spatial coordinates have been annotated in patients schedule).

At least two consecutive MUNE measures were performed on each patient to verify the consistency of our results; when required, further estimations were made until the MUNE was clearly stable. The mean of the two or more tests was calculated [41]. The results showed an excellent reproducibility with test-retest correlation coefficients ranging from 0.75 to 0.86 [32].

### 3.4. Statistical Analysis

Statistics were calculated using a STATISTICA-8^®^ software package [42]. Data were analyzed using a one way ANOVA to compare each other FD and area mean values at different times; a two-way repeated measures ANOVA was performed to assess differences in terms of both FD and area between controls and patients. To isolate which group(s) differ from the others we applied a multiple comparison procedure (Holm-Sidak method). The size of surviving MUs in both biceps brachialis and ADM muscles, expressed as Macro EMG MUP area and peak to peak amplitude, was then compared using the Spearman’s coefficient.

## 4. Conclusions

The main purpose of our study was to assess the extent of MU loss and the changes in the innervation/denervation pattern in fALS compared with sALS patients.

We applied both macro-EMG and MUNE to the BB muscle, and only MUNE to ADM [27]. MUNE is an ideal tool for the assessment of disease in which primary defect is MU loss [29,43]. The reinnervation process is strictly connected with lower motor neuron loss; quantization of MU loss and the simultaneously collateral dynamic re-innervation may be assessed by both MUNE and macro-EMG at every time of disease evolution [30]. The macro-EMG gives a global view of the motor unit. Macro-MUP area and FD were beyond upper normal limits, as expected, in ALS [36,44]. Our results indicate that carriers of SOD-1 mutations have a higher number of motor units at moment of diagnosis when compared with sporadic cases. On the other hand, in sALS the macro-EMG parameters progressively increased, displaying a gradual increment of correlation up to 8 months, suggesting that the process of MU rearrangement begins to fall after 8 months of disease course. In familiar SOD-1 form there is not a specific time interval in which the axonal regeneration and the collateral sprouting can balance the neuronal damage. Paradoxically, despite faster loss of motor units, in fALS we have undisclosed a more effective axonal sprouting in the few surviving motor fibers. Compared with sporadic forms, in SOD-1 fALS the substantial lack of a fleeting stabilization of motor unit number within eight months from clinical onset, as emerged from MUNE, could indicate that damage of cell types different from motor neurons is a critical factor to the progression of corticospinal degeneration [45,46]. Our results strengthen the idea that accelerated disease progression does not alter the timing of disease onset. These data are consistent with those reported by Yamanaka and colleagues [46]: using chimeras derived from embryonic cells of SOD-1^G37R^ mice, they postulated that multiple cell types drive non-cell-autonomous onset of motor degeneration. That could also explain the wide variability in terms of age of onset, clinical presentation and rate of progression in familiar forms of ALS. As to the identity of the cell types beyond motor neurons, potential contributors might include Schwann cells and the endothelial cells of the vasculature. MRI studies have underlined a relative sparing of both cortical and white matter structures in fALS, making unlikely a Wallerian axonal degeneration following primary neuronal injury. This is in line with previous papers showing a differential pyramidal tract degeneration in homozygous SOD-1^D90A^ ALS and sALS [47–49]; e.g., Blain and colleagues have recently reported a marked reduction in fractional anisotropy in the corticospinal tract in patients with sALS and fALS, despite similar levels of upper motor neurons dysfunction and overall clinical disability [47]. Amyotrophic Lateral Sclerosis is featured by repetitive cycles of denervation/reinnervation and the mechanism lead to a variation in FD within a given motor unit [22,50]. SOD-1 carriers had a full complement of motor neurons during the asymptomatic phase, indicating that SOD-1 mutation carriers have normal survival of motor neurons until sudden catastrophic cell death occurs. This significant gradual preclinical loss does not occur in SOD-1 mutation carriers. Despite the small sample of fALS patients, we also tried to detect significant differences in motor unit pool between spinal and bulbar forms, for both BB and ADM muscles. Interestingly, we did not found any difference, suggesting the rate and amount of motor unit decrease is approximately similar in proximal and distal muscles. That could confirm the non length-dependent and all-or-none nature of pathological processes underlying progression of fALS. A possible explanation could be based on an epigenetics approach: it has been proposed that epigenetic silencing of genes vital for motor neuron function could underlie ALS [51,52]. The promoter of genes thought to be implicated in sALS, SOD-1 and VEGF, or that of MT-Ia and MT-II (the most common human isoforms of the metallothionein (MT) family of proteins), has been found with inappropriate methylation levels [53]. There is an increasing interest in this field. Despite this, no conclusive remark has been collected in human models so far. This is likely due to the discrepancy between humans patients and animal models, in terms of disease and pre-symptomatic phase duration, absence of sensitive biological markers and different pathogenesis. Our findings agree with those described by Aggarwal both in symptomatic and asymptomatic SOD-1 mutation carriers: symptomatic fALS could represent an all-or none process and it is not the final result of a slow attrition of motor neurons.

Another interesting finding is about the lack of significant differences in motor unit depletion over time between females and males in SOD-1 type, both in spinal and bulbar form: the antioxidant effects of estrogens and their proved role in preventing glutamate related toxicity *in vitro* [54,55] could not delay both the early retraction of nerve terminals from neuromuscular end-plates and the dying-back of axons during asymptomatic phase *in vivo*, as well as the denervation/reinnervation process at later stages. To an extent, taken together, these results strengthen the idea of a different pathogenetic mechanism at the base of sALS and fALS. Further studies are needed to solve the dilemma, especially in familiar forms different from those related to mutations pertaining to Cu, Zn superoxide dismutase gene.

## Figures and Tables

**Table 1:** Macro-EMG and Motor Unit Number Estimation (MUNE) results, expressed as mean ± 1 SD, both in patients with sALS and fALS and their changes over 4, 8 and 12 months of follow-up.

		T0	T1	T2	T3
		(mean ± 1 SD)	(mean ± 1 SD)	(mean ± 1 SD)	(mean ± 1 SD)
BB Macro EMG	sALS	4397.6 ± 255.9	6389.2 ± 586.3	6553 ± 498.7	8072.4 ± 1023.4
Area (μVms)	fALS	4378.9 ± 319.6	7791.0 ± 953.4	10922.8 ± 1123.7	12499.3 ± 1874.4
BB Macro-EMG	sALS	2.0 ± 0.3	2.1 ± 0.1	2.3 ± 0.3	2.5 ± 0.2
Fiber Density	fALS	1.9 ± 0.3	2.5 ± 0.3	3.5 ± 0.6	3.9 ± 0.5
BB MUNE	sALS	48.3 ± 9.3	34.1 ± 1.9	35.4 ± 1.4	30.8 ± 2.2
	fALS	80.2 ± 7.8	21.8 ± 2.2	16.8 ± 1.0	16.5 ± 2.0
ADM MUNE	sALS	35.6 ± 3.4	20.1 ± 1.8	22.8 ± 5.2	12.1 ± 5.8
	fALS	42.8 ± 6.6	18.4 ± 3.1	15.3 ± 2.1	9.0 ± 2.1
